# Subtotal Cholecystectomy

**DOI:** 10.1155/1996/14515

**Published:** 1996

**Authors:** C. Katsohis, J. Prousalidis, E. Tzardinoglou, A. Michalopoulos, E. Fahandidis, S. Apostolidis, H. Aletras

**Affiliations:** 1 A'Prop. Surg. Clinic Department of Medicine Aristotles University of Thessaloniki AHEPA Hospital Thessaloniki Greece

## Abstract

Subtotal cholecystectomy has been carried out in 34 patients from 1972 to 1992. In the same period 1620 total cholecystectomies were performed. The indications were severe inflammation and/or severe fibrosis in 31 patients, and Mirizzi syndrome type in 3 patients. The morbidity was insignificant, but one patient died, due to severe sepsis. In follow up studies ranging from 6 months to 9 years, there was one patient with retained stones in the common bile duct. No other post cholecystectomy sequelae were noticed in the remaining 32 patients. Subtotal cholecystectomy is a safe, feasible and definitive operation in patients for whom the standard operation could be dangerous. This operation is less burdensome to the patient, and is accompanied by fewer complications than ordinary cholecystostomy.


*HPB Surgery*, 1996, Vol. 9, pp.133–136
Reprints available directly from the publisher
Photocopying permitted by license only

© 1996 OPA (Overseas Publishers Association)
Amsterdam B.V. Published in The Netherlands
by Harwood Academic Publishers GmbH
Printed in Malaysia

### Subtotal Cholecystectomy

C. KATSOHIS, J. PROUSALIDIS, E. TZARDINOGLOU, A. MICHALOPOULOS
E. FAHANDIDIS, S. APOSTOLIDIS and H. ALETRAS

A'Prop. Surg. Clinic, Department of Medicine, Aristotles University of Thessaloniki AHEPA Hospital, Thessaloniki, Greece

*(Received 30 June 1993)*

Subtotal cholecystectomy has been carried out in 34 patients from 1972 to 1992. In the same period 1620 total cholecystectomies were performed. The indications were severe inflammation and/or severe fibrosis in 31 patients, and Mirizzi syndrome type 1 in 3 patients. The morbidity was insignificant, but one patient died, due to severe sepsis. In follow up studies ranging from 6 months to 9 years, there was one patient with retained stones in the common bile duct. No other post cholecystectomy sequelae were noticed in the remaining 32 patients. Subtotal cholecystectomy is a safe, feasible and definitive operation in patients for whom the standard operation could be dangerous. This operation is less burdensome to the patient, and is accompanied by fewer complications than ordinary cholecystostomy.

KEY WORDS: Cholecystectomy Mirizzi Syndrome type 1

Cholecystectomy is a common but potentially difficult operation because of the variable anatomy, the inflammation and fibrosis present in the wall of gallbladder and the surrounding tissues and possible coexistent medical problems such as portal hypertension. At times it can challenge even the most talented surgeon.

However, recent developments in laparoscopic cholecystectomy have given a new impetus to the traditional operation [1]. It is also clear that patients now tend to be older, and the surgical mortality rate is higher in this group reaching, for patients over 80 years of age, 7 per cent for elective cholecystectomy and 14 per cent if the operation is perfromed for acute cholecystitis[2,3].

Therefore a number of alternatives to cholecystectomy have been proposed to eliminate stones without removing the gallbladder. Oral dissolution agents (chenodeoxycholic and ursodeoxycholic acid)[4], direct gallstone dissolution with methyl tert-butyl ether[5] and extracorporeal shockwave lithotripsy (ESWL)[6] have been used. These alternatives to cholecystectomy are effective in selected groups of patients and clearance of stones can be accompanied by relief of symptoms[7]. Cholecystolithotomy instead of cholecystectomy has been tried, but it is seldom employed because stone formation can recur. However, using new endoscopic and radiological techniques it is possible to ensure complete clearance of the gallbladder after cholecystolithotomy. Following cholecystostomy and removal of calculi, 37–83 per cent of patients develop recurrent stones [8,9] mainly from fragments or stones overlooked at the initial operation.

The presence of portal hypertension on the other hand increases the danger of cholecystectomy. In these high-risk patients the reported mortality rate is 83%[10]. It has therefore been suggested that the indications for elective cholecystectomy in patients with cirrhosis should be restricted and that the operation should be performed only where there are life-threatening complications[10].

Subtotal cholecystectomy has been described as an easy, safe and definitive alternative to standard cholecystectomy in a number of situations including portal hypertension[11]. For patients with the Mirizzi syndrome type 1 recent publications recommend subtotal cholecystectomy as the operation of choice[12].

This report reviews a series of 34 subtotal cholecystectomies performed from 1972 to 1992. The purpose of this study is to assess the results of the procedure and to increase awareness of the operation as an alternative to standard cholecystectomy or cholecystostomy, with which it is compared.

*Correspondence to*: C. Katsohis. M.D. Professor of Surgery A' Prop. Surgical Clinic, AHEPA Hospital, Stilp. Kiriakidi 1, 54636 Thessaloniki, Greece.

### MATERIALS AND METHODS

From 1972 to 1992, 1740 patients were subjected to billiary surgery in the A.Propedeutic Surgical Clinic, Medical School of Aristotles University, AHEPA Hospital, Thessaloniki, Greece. Of them 1621 received simple cholecystectomy, and 85 cholecystostomy, whereas 34 patients were subjected to subtotal cholecystectomy (Table 1). The age of the patients of the present series ranged from 40 to 81 years (mean 61.5); 16 were males and 18 females. They were hospitalized from 7 to 35 days, (mean 13.5 days). One patient was hospitalized for 135 days because of continuous bile leakage. The indications in the present series were: severe inflammation and fibrosis (31 patients), and Mirizzi syndrome type 1 (3 patients) (Table 2). In these cases dissection of the area of Calot was found to be difficult and in most instances dangerous.

Fifteen patients were subjected to subtotal cholecystectomy only. Nine patients, after completion of subtotal cholecystectomy, underwent biliary tree imaging via a patent cystic duct. Four patients received subtotal cholecystectomy and drainage performed via Hartman's pouch. Common bile duct exploration with T-tube placement, after subtotal cholecystectomy was carried out in another four cases and in two cases choledochoduodenal anastomosis was performed. In all cases a subhepatic drain was left in place (Table 3).

**Table 1** Operations performed for cholelithiasis from 1972 to 1992

| *Operations* | *Patients* | *per cent* |
|---|---|---|
| Total cholecystectomies | 1621 | 93.12 |
| Cholecystostomies | 85 | 4.88 |
| Subtotal cholecystectomies | 34 | 2 |
| Total | 1740 | 100.00 |

**Table 2** Indications for subtotal cholecystectomy

| *Condition or Disease* | *Patients* |
|---|---|
| Inflammation ± Fibrosis | 31 |
| Urgent cholecystectomy | 12 |
| Elective surgery | 15 |
| Jaundice | 3 |
| Bile peritonitis | 1 |
| Mirizzi syndrome type 1 | 3 |
| Total | 34 |

The indications for operative x-ray investigation were based on history (two with jaundice), ultrasonographic data (stones in the biliary tree, duct dilatation), small stones in the gallbladder, dilated common bile duct, operational difficulties and for delineating the anatomy.

**Table 3** Operations performed and technical specifications

| *Operations* | *patients* |
|---|---|
| -S.C. only | 15 |
| -S.C. and biliary tree imaging via a patent Cystic duct | 9 |
| -S.C. and drainage via the Hartman's pouch | 4 |
| -S.C. and CBD exploration with T-tube placemen | 4 |
| -S.C. and choledochoduodenal anastomosis | 2 |
| Total | 34 |

In all cases a subhepatic drain was left in place
S.C.: Subtotal cholecystectomy, CBD: Common bile duct

In patients with Mirizzi syndrome type 1 the diagnosis was made during the operation. All patients were put on antibiotic therapy which was continued in 21 of them according to the inflamation encountered (mean duration of antibiotic therapy, 7 days).

All patients were followed up from 6 months to 9 years.

### OPERATIVE TECHNIQUE

A light subcostal or paramedian incision was used. In all cases the area of Calot's triangle was inspected and the decision to perform subtotal rather than a total cholecystectomy was taken according to the difficulties encountered. The gallbladder was usually opened at the fundus or at a convenient site, evacuated with the bile being sent for culture. The gallbladder was then excised using diathermy, leaving in situ the wall of that portion of the gallbladder directly in contact with the liver bed and Calot's triangle. The mucosa was cauterized with electrodiathermy. Portion of the Hartman's pouch was left intact without identifying the structures in the triangle of Calot.

In patients with an occluded cystic duct and in the cases of extensive inflammation/fibrosis and Mirizzi syndrome, no attempt was made to perform cholangiography. Puncture of the common bile duct was avoided. In those patients in whom the cystic duct was found patent from within the Hartman's pouch, cholangiography was enabled by passing the catheter into the duct. The catheter was usually held in place by a purse-string like suture within Hartman's pouch.

In 9 cases in the present series the cystic duct was patent and an operative cholangiography was carried out. In another 4 cases a catheter was placed in Hartman's pouch for draining and postoperative cholangiography. In 4 more patients exploration of the common bile duct was carried out and a T-tube left in place. In two patients with common bile duct stones a choledochoduodenal anastomosis was performed.

Upon completion of subtotal cholecystectomy, the patent cystic duct was sewn from within the gallbladder remnant with a continuous 3/0 silk or Vicryl suture. The gallbladder area was drained using a suction drain, or a Penrose Latex drain.

Whenever visualization of the biliary tree was considered necessary, a postoperative endoscopic retrograde cholangiography was carried out.

### RESULTS

Thirty four patients underwent subtotal cholecystectomy from 1972 to 1992. This represents 2% of the 1620 cholecystectomies and 85 cholecystostomies, performed during the above period (Table 1).

Three patients underwent endoscopic retrograde cholangiography postoperatively for raised alkaline phosphatase or dilatation of the common bile duct found on ultrasonography. In 1 of them endoscopic sphincterotomy was carried out postoperatively, because of retained stones. In 4 cases postoperative cholangiography was carried out through the T-tube. In 4 patients cholangiography through the catheter in the Hartman's pouch was carried out (Table 3).

Two patients developed wound infection (5.2 percent), and five more patients developed a bile leak from the drain which gradually stopped, without surgical intervention (14.7 percent). One patient in the present series died from overwhelming sepsis caused by bile peritonitis (Table 4). All patients followed up from 6 months to 9 years, were free of symptoms.

### DISCUSSION

Subtotal cholecystectomy is a safe, satisfying procedure in patients for whom the standard operation would entail considerable danger[11]. The possibility of damage in the region of Calot due to distorted anatomy caused by extensive fibrosis and severe inflammation can be ablated by this procedure. The possibility of damage to the common bile duct, common and right hepatic ducts and the right hepatic artery, is high in such cases. In the Mirizzi syndrome type 1, Hartmann's pouch with its contained stone is adhered to the common hepatic duct as a result of long-standing inflammation and fibrosis, and Calot's triangle is obliterated. In such cases, dissection of the gallbladder from the common hepatic duct is difficult and hazardous and quite often leads to damage of the duct. Baer *et al.*[12] have suggested that subtotal cholecystectomy is the treatment of choise for the Mirizzi syndrome type 1.

Imaging of the biliary tree, even though problematic in these cases, is most helpful pre-,during, or postoperatively in an effort to delineate the anatomy and look for remaining stones etc. Operative cholangiography when the cystic duct is patent, and pre or postoperative ERCP when the cystic duct is obliterated, are recommended. Direct puncture of the common bile duct is hazardous. In jaundiced patients these examinations, with the addition of percutaneous transhepatic cholangiography are of upmost importance. In patients suspected pre-operatively of having the Mirizzi syndrome, endoscopic retrograde cholangiography is highly advocated[13].

Cholecystectomy in the presence of portal hypertension is a hemorrhagic operation, followed by high mortality rate in spite of the preoperative correction of coagulopathy. Abnormalities of liver function, often assosiated with jaundice, require differential diagnosis between common bile duct stones and hepatocellular disease. To define the cause of jaundice in these situations ERCP is most helpful, with the addition of endoscopic sphincterotomy when needed. Subtotal cholecystectomy solves this problem.

Cholecystostomy as an alternative to cholecystectomy has the disadvantage of a high rate of retained stones (27 to 75% according to various authors)[14]. Winkler et al, have reported a mortality rate of 5 per cent with cholecystostomy, but 70 per cent of their surviving patients underwent cholecystectomy 6-8 weeks later[15].

In conclusion subtotal cholecystectomy avoids the need for a second operation, is an easy and safe procedure, with minor complications, low mortality rate, and must be considered when cholecystectomy appears hazardous.

**Table 4** Complications

| | *Patients* | *Per cent* | *Remarks* |
|---|---|---|---|
| Wound infections | 2 | (5.2%) | |
| Bile leak (around the drain) | 5 | (14.7%) | |
| Retained CBD stones | 1 | (2.9%) | |
| Bile peritonitis | 1 | (2.9%) | Death |

### REFERENCES

1. Reddick EJ, Olsen DO.(1989) Laparoscopic laser cholecystectomy. *Surg Endosc* **3**: 131–133.
2. Editorial.(1989) Gallstones, bile acids and the liver. *Cancer* **II**: 249–251.
3. Gutman H. Deutsch AA, Nudelman IL, Reiss R. (1988) Cholecystectomy for octogenarians *Dig Surg* **5**: 189–193.
4. Fromm H. (1986) Gallstone dissolution therapy: current status and future prospects. *Gastroenterology* **91**: 1560–1567.
5. Thistle JL.(1987) Direct contact dissolution of gallstones. *Semin Liver Dis* **7**: 311–316.
6. Darzi A, Monson JRT, O'Morain C, Tanner WA, Keane FBV.(1989) Extension of selection criteria for extracorporeal shockwave lithotripsy. *Br Med J* **299**: 302–303.
7. Darzi A, Leahy AL, Feeley T, Tanner WA, Keane FBV.(1989) Symptomatology of patients post gallstone clearance in patients receiving ESWL. *Gut* **30**: A1508.
8. Schildt E.(1960) On cholecystolithotomy. *Acta Soc Med Uppsala* **65**:91–95.
9. Norrby F, Schonebeck J. (1970) Long-term results with cholecystolithotomy. *Acta Chir Scand* **136**: 711–713.
10. Aranha GV, Sontag SJ, Greenlee HB.(1982) Cholecystectomy in cirrhotic patients a formidable operation. *Am J Surg* **143**: 55–60.
11. Bornman PC, Terlanche J. (1985) Subtotal cholecystectomy:for the difficult gallbladder in portal hypertension and cholecystitis. *Surgery* **98**:1–6.
12. Baer HU, Matthew JB, Schweizer WP, Gertsch P, Blumgart LH.(1990) Management of the Mirizzi syndrome and the surgical implication of cholecystcholedochal fistule. *Br J Surg* **77**: 743–745.
13. Cottier DJ, McKay C, Anderson JR.(1991) Subtotal Cholecystectomy. *Br J Surg* **78**: 1326–1328.

